# Wearable inertial sensors are highly sensitive in the detection of gait disturbances and fatigue at early stages of multiple sclerosis

**DOI:** 10.1186/s12883-021-02361-y

**Published:** 2021-09-04

**Authors:** Roy Müller, Daniel Hamacher, Sascha Hansen, Patrick Oschmann, Philipp M. Keune

**Affiliations:** 1 GaitLab, Klinikum Bayreuth GmbH, Bayreuth, Germany; 2 Department of Neurology, Klinikum Bayreuth GmbH, Bayreuth, Germany; 3 Department of Sports Science, Friedrich Schiller University Jena, Jena, Germany; 4 Institute of Psychology, Otto-Friedrich-University, Bamberg, Germany

**Keywords:** Multiple sclerosis (MS), 6-min walk, 25 foot walk, toe clearance, motor fatigue, EDSS

## Abstract

**Background:**

The aim of the current study was to examine multiple gait parameters obtained by wearable inertial sensors and their sensitivity to clinical status in early multiple sclerosis (MS). Further, a potential correlation between gait parameters and subjective fatigue was explored.

**Methods:**

Automated gait analyses were carried out on 88 MS patients and 31 healthy participants. To measure gait parameters (i.e. walking speed, stride length, stride duration, duration of stance and swing phase, minimal toe-to-floor distance), wearable inertial sensors were utilized throughout a 6-min 25-ft walk. Additionally, self-reported subjective fatigue was assessed.

**Results:**

Mean gait parameters consistently revealed significant differences between healthy participants and MS patients from as early as an Expanded Disability Status Scale (EDSS) value of 1.5 onwards. Further, MS patients showed a significant linear trend in all parameters, reflecting continuously deteriorating gait performance throughout the test. This linear deterioration trend showed significant correlations with fatigue.

**Conclusions:**

Wearable inertial sensors are highly sensitive in the detection of gait disturbances, even in early MS, where global scales such as the EDSS do not provide any clinical information about deviations in gait behavior. Moreover, these measures provide a linear trend parameter of gait deterioration that may serve as a surrogate marker of fatigue. In sum, these results suggest that classic timed walking tests in routine clinical practice should be replaced by readily and automatically applicable gait assessments, as provided by inertial sensors.

## Background

Multiple sclerosis (MS) is often associated with a decline in walking ability and balance control [1–6]. Moreover, around 40 % of people with MS report walking problems that negatively affect their quality of life [7].

To measure walking ability in MS patients, a wide range of tests is available. The most commonly used tests are the timed 25-foot walk, that measures the time it takes a patient to walk a 25 feet distance as fast as possible, or the 6-min walk, that measures the total distance a patient can walk in six minutes. During these walking tests, MS patients typically display a significantly lower mean walking speed compared to healthy participants [8–11]. However, parameters that are usually derived from these walking tests (i.e. average speed or total distance) are not suitable to study walking characteristics that may vary over time of continuous physical activity.

Several studies have examined dynamic walking characteristics, such as the progression of walking speed throughout the test duration [1, 8]. For example, Goldman et al. examined walking speed profiles in MS patients during the 6-min walk and found that MS patients differed from healthy participants in both, the mean walking speed and the course of walking speed across the 6-min time span (calculated per minute) [8]. The latter revealed that patients decelerate continuously across the 6-min observation period, yielding a significant linear trend component. Additionally, according to the results of Goldman et al., the 6-min walk distance also distinguished MS patients based on their Expanded Disability Status Scale (EDSS [12]), i.e. patients with mild disability (EDSS 0–2.5) showed a similar pattern to healthy participants whereas patients with moderate (EDSS 3–4) and severe (EDSS 4.5–6.5) disability displayed a deceleration throughout the walking test. Based on these findings Burschka et al. examined a potential association between the linear deceleration trend during both a 6-min and a 12-min walking test on the one hand and self-reported fatigue on the other hand [1]. Results revealed that the linear deceleration trend was highly correlated with subjective fatigue. Moreover, the linear trend component was superior in predicting subjective fatigue, as compared to average walking speed.

Results of both Goldman et al. [8] and Burschka et al. [1] revealed that MS patients decelerate continuously across the 6-min observation period. However, the extent to which the reduction in walking speed was caused by e.g. decreasing stride lengths and/or increasing contact times was not investigated. Therefore, the functional mechanisms underlying the linear deceleration trend remain to be explored in detail. With the appearance of electronic walkways and wearable inertial sensors, it has become possible to measure additional gait parameters in a clinical setting, e.g. step length and width or step time [6, 9, 10, 13–15]. For example, Socie et al. examined temporal gait parameters during the 6-min walk in MS patients and healthy participants using an electronic walkway [9]. They found that MS patients had a significantly greater reduction in walking speed over the course of the 6-min walk, which coincided with a significantly greater increase in step time and double support. Comparable results can be observed in gait analysis using wearable inertial sensors [6, 14–16]. However, to the best of our knowledge, the progression of distinct temporal gait parameters throughout the test duration, as obtained by wearable inertial sensors, has not been investigated so far. This appears striking as Burschka et al. reported that particularly the linear trend components of classical walking parameters (e.g. linear deceleration during a walking test) were predictive of self-reported fatigue [1]. It may be assumed that an automatic assessment using wearable inertial sensors in combination with a model that is highly predictive of fatigue (linear trend component) may yield a useful tool for standardized clinical assessments addressing symptoms of motor fatigue in MS.

The purpose of the current study was to examine multiple gait parameters (i.e. mean gait parameters and linear trend components) obtained by means of wearable inertial sensors and their sensitivity to patients’ clinical status based on their EDSS. Further, we collected self-report data about somatic fatigue, in order to verify whether walking dynamics were related to patient’s subjective constraints.

## Methods

### Participants

MS patients and healthy participants were recruited in the Department of Neurology of the Klinikum Bayreuth GmbH, Germany. Patients were eligible to participate in case of a verified MS diagnosis [17], or clinically isolated syndrome (CIS), an age between 18 and 65 years and the ability to walk without a walking aid for at least six minutes. Patients were not included in case of a recent treatment change or relapse.

An a priori power analysis for an ANOVA model conducted by means of G*Power 3.1.5 software revealed the necessity of 128 participants, given the following input parameters: effect size F = 0.3 (detectable), alpha error probability: 0.05, power: 0.8, and number of groups: 4 (healthy comparison group, MS group 1 (EDSS 0.0–1.0), MS group 2 (EDSS 1.5-2.0), MS group 3 (EDSS 2.5-5.0)). From the 128 recruited participants *N* = 119 datasets were available for the final analysis, involving *N* = 88 MS patients and *N* = 31 healthy participants (see Table 1 for details and distribution across groups). A post hoc analysis revealed that with the available sample size of *N* = 119 and constant alpha error probability (0.05) and power (0.08), the final detectable effect remains almost unchanged. Given that previous work examining linear gait trend components in MS reported compatible observable effects [3], the sample size of the current study may be regarded as appropriate.

**Table 1:** Demographical and clinical characteristics of the sample

	Comparison group	MS group 1 (EDSS 0.0–1.0)	MS group 2 (EDSS 1.5-2.0)	MS group 3 (EDSS 2.5-5.0)
Participants number	31	27	29	32
Female sex	22	20	23	21
Age [years]	34.6 ± 8.8	37.9 ± 10.4	38.3 ± 11.2	47.9 ± 9.7^a,b,c^
Height [cm]	173.2 ± 8.6	172.0 ± 7.2	169.7 ± 7.0	171.5 ± 8.6
Weight [kg]	71.4 ± 11.5	76.4 ± 18.3	80.0 ± 19.3	79.3 ± 17.8
EDSS	NA	0.8 ± 0.4	1.9 ± 0.2	3.1 ± 0.6
Type of MS				
clinically isolated syndrome	NA	1	0	0
Relapsing-remitting	NA	26	29	26
Secondary progressive	NA	0	0	5
Primary progressive	NA	0	0	1

Values of age, height, weight and EDSS are expressed as mean ± SD. *MS* Multiple Sclerosis; *EDSS* Expanded Disability Status Scale. Significant differences from healthy controls, MS group 1 and MS group 2 are indicated with ‘a’, ‘b’, and ‘c’, respectively (*p* < 0.05)

All participants provided written informed consent. The study was approved by the ethical review board of the Friedrich Schiller University Jena, Germany (2018-1221-BO) and was in accordance with the Declaration of Helsinki.

### Measurements

To measure gait parameters (i.e. walking speed, stride length, stride duration, the duration of the stance and swing phase as well as the minimum toe-to-floor distance), wearable inertial sensors were utilized (MTw2, Xsens Technologies B.V.; sampling rate: 100 Hz) throughout the walking course of a 25-foot distance. The sensors were attached to the forefoot of participants’ dominant leg (i.e., the foot they would take to kick a ball).

Assessments took place in the Klinikum Bayreuth GmbH, Department of Neurology. Both MS patients and healthy participants had to complete a walking test that required them to cover a distance of 25 feet repeatedly throughout a maximal assessment period of six minutes as enduring and fast as possible (6-min 25-ft walk [3, 6]). A cone was placed three feet away from each endpoint of the 25-foot distance and participants circle the cones to make their turn back toward the 25-foot distance. In addition to the walking test and gait parameter measures, observer-rater tests (Berg Balance Scale, BBS [18] and Timed-up and Go Test, TUG [19]) as well as a self-report measure addressing fatigue (WEIMuS [20, 21]) was administered.

### Data processing

To exclude effects of acceleration and deceleration the first and the last 25 feet distance, as well as the first and the last 2.5 m of each 31 feet distance between the cones were excluded from the following analysis [6, 22]. To calculate gait parameters (i.e. walking speed, stride length, stride duration, the duration of the stance and swing phase as well as the minimum toe-to-floor distance), a validated algorithm was used [23, 24]. Heel strikes and toe-off events were identified based on local minima of the angular velocity of the foot in the sagittal plane. In addition, for all participants, the linear trend components (slope of the regression line) of all gait parameters were determined across all included strides measured during each minute of the walking test.

Statistical analyses were performed with SPSS 20 (Chicago, IL, USA). To test normality of distributions, Kolmogorov-Smirnov tests were implemented for all gait parameters. Differences in gait parameters and linear trend components between MS patients and healthy participants were assessed by a one-way between-subjects ANOVA (factor group: MS group 1–3, healthy comparison group) with post-hoc analysis. Linear and quadratic trends of the gait parameters were assessed by a two way repeated measures ANOVA with the within-subjects factor minute (1, 2, 3, 4, 5, 6) and the between-subjects factor group (MS group 1–3 and healthy comparison group). This model tested whether gait parameters varied throughout the test and across groups. To examine the assumed association between gait parameters and subjective fatigue, Pearson correlation coefficients were calculated.

## Results

### Mean gait parameters

**Fig. 1:**
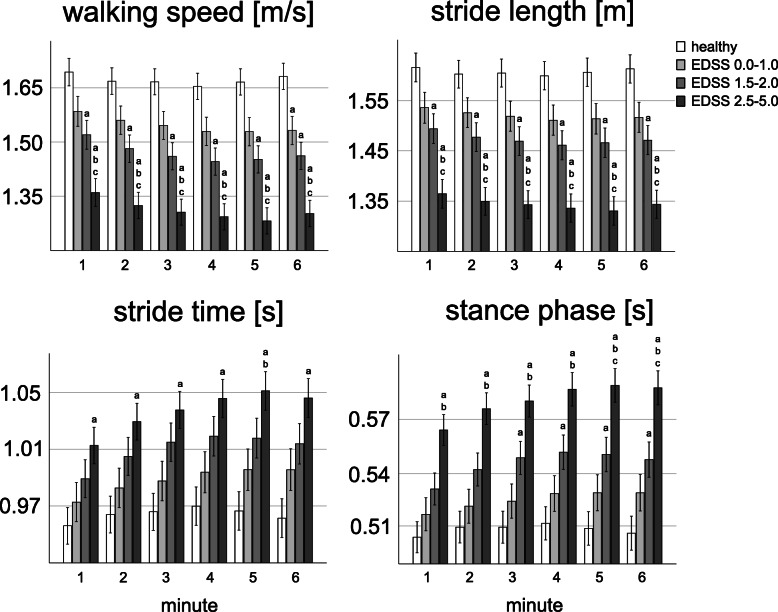
Walking speed, stride length, stride time and duration of the stance between healthy participants and MS patients (MS group 1: EDSS 0.0–1.0, MS group 2: EDSS 1.5-2.0, MS group 3: EDSS 2.5-5.0), separated for each minute. Error bars represent standard error. Significant differences from healthy controls, MS group 1 and MS group 2 are indicated with ‘a’, ‘b’, and ‘c’, respectively (*p* < 0.05).

**Fig. 2:**
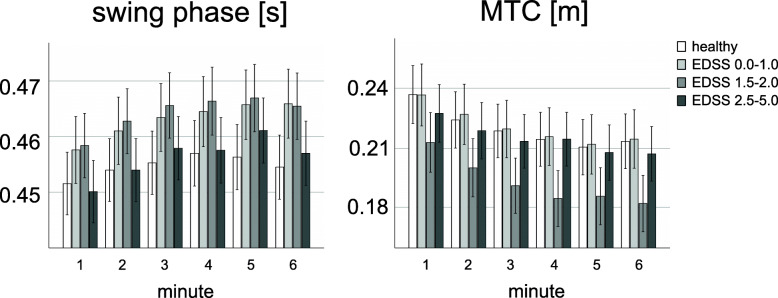
Duration of the stance phase and minimum toe-to-floor distance (MTC) between healthy participants and MS patients (MS group 1: EDSS 0.0–1.0, MS group 2: EDSS 1.5-2.0, MS group 3: EDSS 2.5-5.0), separated for each minute. Error bars represent standard error. Significant differences from healthy controls, MS group 1 and MS group 2 are indicated with ‘a’, ‘b’, and ‘c’, respectively (*p* < 0.05).

All gait parameters (except stance phase time in MS group 2) were normally distributed in the healthy comparison group and MS group 1–3. Compared to healthy participants, MS patients walked slower, took shorter stride lengths and took more time to take the strides (Table 2; Fig. 1). The increased stride duration in MS patients was attributable to an increased stance phase time (the swing phase time remained unchanged; Table 2; Fig. 2). Post-hoc comparisons of the mean gait parameters indicated significant differences between healthy participants and MS patients from MS group 2 (EDSS 1.5-2.0) onwards. For example, the mean stride length in MS group 1 (EDSS < 1) was decreased by about 6 % (p = 0.229) relative to the healthy comparison group. In MS group 2 this decrease was more pronounced, i.e. about 9 % (p = 0.008) and in MS group 3 (EDSS > 2) about 17 % (*p* < 0.001). The minimum toe-to-floor distance did not differ between healthy participants and MS patients (Table 2; Fig. 2).

**Table 2:** Parameters of gait, observer-rater tests and fatigue

	Comparison group	MS group 1 (EDSS 0.0–1.0)	MS group 2 (EDSS 1.5-2.0)	MS group 3 (EDSS 2.5-5.0)
**mean gait parameter**				
walking speed [m/s]	1.67 ± 0.18	1.55 ± 0.17	1.47 ± 0.19 ^a^	1.30 ± 0.25 ^a,b,c^
stride length [m]	1.61 ± 0.16	1.52 ± 0.13	1.47 ± 0.13 ^a^	1.33 ± 0.20 ^a,b,c^
stride time [s]	0.96 ± 0.06	0.99 ± 0.07	1.01 ± 0.07	1.04 ± 0.09 ^a^
stance phase [s]	0.51 ± 0.03	0.53 ± 0.04	0.55 ± 0.05 ^a^	0.58 ± 0.07 ^a,b^
swing phase [s]	0.45 ± 0.03	0.46 ± 0.03	0.46 ± 0.03	0.46 ± 0.05
MTC [cm]	2.2 ± 0.6	2.2 ± 0.7	1.9 ± 0.5	2.1 ± 1.1
**linear trend of mean gait parameter**				
walking speed slope	-0.002 ± 0.016	-0.011 ± 0.014	-0.011 ± 0.014	-0.012 ± 0.014 ^a^
stride length slope	-0.000 ± 0.009	-0.004 ± 0.007	-0.004 ± 0.008	-0.005 ± 0.008
stride time slope	0.001 ± 0.004	0.005 ± 0.005	0.005 ± 0.005	0.007 ± 0.008 ^a^
stance phase slope	0.000 ± 0.003	0.002 ± 0.003	0.003 ± 0.003 ^a^	0.005 ± 0.006 ^a^
swing phase slope	0.001 ± 0.002	0.002 ± 0.002	0.001 ± 0.002	0.002 ± 0.002
MTC slope	-0.001 ± 0.000	-0.001 ± 0.001	-0.001 ± 0.000	-0.000 ± 0.000
**observer-rater tests**				
BBS	56.0 ± 0.0	55.7 ± 1.2	55.0 ± 2.5	51.8 ± 4.8^a,b,c^
TUG [s]	4.5 ± 0.6	5.2 ± 1.0	5.8 ± 1.3^a^	7.0 ± 1.6^a,b,c^
**fatigue**				
WEIMus	2.2 ± 4.0	6.7 ± 7.1	9.6 ± 7.1 ^a^	17.7 ± 8.3 ^a,b,c^

Values are expressed as mean ± SD. *MTC* minimum toe-to-floor distance. Significant differences from healthy controls, MS group 1 and MS group 2 are indicated with ‘a’, ‘b’, and ‘c’, respectively (*p*<0.05)

### Linear trend component

In contrast to healthy participants, MS patients showed a significant linear trend in all gait parameters during the 6-min 25-ft walk (Figs. 1 and 2). In particular, walking speed (F(1,84) = 60.12, p = 0.000), stride length (F(1,84) = 27.95, *p* = 0.000) and minimum toe-to-floor distance (F(1,84) = 84.98, *p* = 0.000) decreased, whereas stride time (F(1,84) = 68.62, p = 0.000), stance phase time (F(1,84) = 55.12, p = 0.000) and swing phase time (F(1,84) = 51.22, *p* = 0.000) increased. The linear trend was not differentially expressed across the MS groups. However, as revealed by a significant minute by group interaction the linear trend in walking speed (F(3,114) = 3.24, *p* = 0.025), stride time (F(3,114) = 5.54, *p* = 0.001) and stance phase time (F(3,114) = 6.41, *p* = 0.000) across all groups (MS groups and healthy comparison group) was differentially expressed.

### Quadratic trend component

Similar to healthy participants, MS patients showed a quadratic trend in all gait parameters during the 6-min 25-ft walk (Figs. 1 and 2). In particular, MS patients showed a U-shaped profile in walking speed (F(1,84) = 91.68, *p* = 0.000), stride length (F(1,84) = 44.79, *p* = 0.000) and minimum toe-to-floor distance (F(1,84) = 13.60, *p* = 0.000), and an inverse U-shaped profile in stride time (F(1,84) = 66.06, *p* = 0.000), stance phase time (F(1,84) = 62.43, *p* = 0.000) and swing phase time (F(1,84) = 34.44, *p* = 0.000). As revealed by a significant minute by group interaction, the quadratic trend in stride time and stance phase time was differentially expressed across MS groups (stride time: F(2,84) = 3.14, *p* = 0.048, stance phase time: F(2,84) = 4.94, *p* = 0.009) and in stance phase time across all groups (F(3,114) = 4.22, *p* = 0.007).

### Observer-rater tests and fatigue

Of the 88 MS patients who took part in the walking test, 70 patients (see Table 3) also completed the self-report measure addressing fatigue. Post-hoc comparisons of the mean observer-rater tests (BBS, TUG) and subjective fatigue indicated significant differences between healthy control participants and MS patients (Table 2). Within the MS group, the BBS score decreased (*r* = 0,433, *p* = 0,000) and the time for the TUG (*r* = 0,495, *p* = 0,000) and the somatic fatigue (*r* = 0,525, *p* = 0,000) score increased with disease progression. Furthermore, within the MS group, linear trend components of BBS score, walking speed, stride length, stride time and stance phase time showed significant correlations with fatigue in MS group 3 (Table 3).

**Table 3:** Correlation coefficients

	MS group 1 (EDSS 0.0–1.0)	MS group 2 (EDSS 1.5-2.0)	MS group 3 (EDSS 2.5-5.0)
	WEIMus		
**mean gait parameter**			
walking speed [m/s]	0.013	-0.406	-0.364
stride length [m]	0.123	-0.388	-0.325
stride time [s]	0.088	0.251	0.366
stance phase [s]	-0.014	0.353	0.161
swing phase [s]	0.203	0.013	0.372
MTC	0.150	-0.103	-0.213
**linear trend of mean gait parameter**			
walking speed slope	-0.003	0.144	-0.477^a^
stride length slope	-0.020	0.251	-0.443^a^
stride time slope	0.073	-0.051	0.428^a^
stance phase slope	0.066	0.042	0.403^a^
swing phase slope	0.009	-0.186	0.260
MTC slope	-0.074	0.194	-0.121
**observer-rater tests**			
BBS	-0.357	-0.374	-0.462^a^
TUG	-0.068	0.131	0.127
*N*	*23*	*22*	*25*

^a^correlation is significant at the 0.05 level (2-tailed)

## Discussion

### Gait parameter during the 6-min 25-ft walk

The results of this study indicate that wearable inertial sensors can be used as a suitable measuring instrument for recording mean gait parameters (and linear trend components) in MS patients. This is in accordance with previous studies [6, 14, 15, 25]. Moreover, the mean gait parameters measured in our examination (during the 6-min 25-ft walk) are also comparable to other studies using a stop-watch [1, 8] or an electronic walkway [9, 26]. For example, in Burschka et al. [1] mean walking speed during the 6-min walk decreased from 1.89 m/s for healthy participants to 1.63 m/s for mildly disabled MS patients (EDSS 0-3.5) and to 1.17 m/s for moderately disabled MS patients (EDSS 4–5) and in Goldman et al. [8] walking speed decreased from 1.68 m/s for healthy participants to 1.64 m/s for MS patients with EDSS 0-2.5, to 1.36 m/s for MS patients with EDSS 3–4 and to 1.05 m/s for MS patients with EDSS 4.5–6.5. Due to the reduced walking speed in MS patients with EDSS 0-2.5 [8], the 6-min walk test seems to be appropriate to evaluate differences between mildly disabled MS subjects and healthy participants. However, compared to both Goldman et al. and Burschka et al. our classification of MS patients was more differentiated in the early stages of MS. This made it possible to find significant differences in mean walking speed from MS group 2 (EDSS 1.5-2) onwards. In addition to walking speed, comparable differences from MS group 2 onward can also be seen in other mean gait parameters (i.e. stride length, stance phase time). Thus, we suggest that these mean gait parameters (measured with wearable inertial sensors) are suitable for separating MS patients, even in the early stages of MS (EDSS > 1.5, see Table 2), in which the EDSS may not provide information about deviations in gait behavior.

In contrast to walking speed, stride length and stance phase time, other mean gait parameters such as minimum toe-to-floor distance (MTC) and swing phase time do not appear to be suitable for distinguishing MS patients with EDSS score of less than 5. A closer look at the MTC reveals that in MS group 2 mean MTC and standard deviation of MTC decreases (Table 2) and in MS group 3 standard deviation of MTC increases (Table 2) compared to both healthy comparisons and MS group 1. However, differences were not significantly. In a review of Barrett et al., it was suggested that a higher MTC variability would increase the risk of tripping in older adults [27]. Thus, we suggest that MTC could be a valuable indicator for fall risk in MS patients. However, it is very speculative and needs to be proven in further studies.

When comparing mean gait parameters with observer rater tests comparable differences from MS group 2 onward can also be seen in TUG (Table 2). During the TUG, time required to stand up from sitting, walk a distance of three meters and return to a chair and sit back down again was recorded [19]. The time to complete the task (from signal to start to the moment the participant’s body returns to the seat pan of the chair) is measured with a stop-watch and thus, in part depending on the subject who measures the time. Automatically applicable gait assessments, as provided by inertial sensors, provide more objective results.

### Linear trend of gait parameter and fatigue

More than a third of MS patients experience walking-related motor fatigue during the 6-min walk, with the prevalence being highest in more disabled patients [28]. Therefore, the identification of motor fatigue associated with walking is of great interest. With the appearance of electronic walkways and wearable inertial sensors, it became possible to measure multiple gait parameters and their progression throughout a walking test. Our results show that in contrast to the healthy comparison group MS patients depict a linear trend in all gait parameters throughout the 6-min 25-ft walk (Fig. 1; Table 2). However, the linear trend was not differentially expressed across the MS groups. Hence, it seems that the linear trend (in contrast to the mean gait parameter) is not sensitive to differentiate MS patients with mild disability.

In addition to the walking test, we administered a self-report measure addressing fatigue. Our results show that the somatic fatigue score increased with disease progression (based on EDSS) and that somatic fatigue indicated significant differences between healthy control participants and MS patients (Table 2). However, significant correlations between fatigue and measured gait parameters can be found for linear trend components (in MS group 3) but not for mean gait parameters. Thus, we suggest that the linear trend (and not the mean) of measured gait parameters (i.e. slope of walking speed, stride length, stride time and/or stance phase time; Table 3) can be used as a good predictor for somatic fatigue.

### Limitations of the study

Some limitations of the present study require consideration. First, the mean age for MS group 3 was almost 10 years higher than the other groups (Table 1). Since gait parameters (e.g. walking speed and stride length) change with age [29], some of the differences between MS group 3 and the other groups can be explained by age-related effects. However, there was no significant difference in age between MS group 2 and healthy participants but significant differences in walking speed, stride length and stance phase time (Table 2). Thus, age is probably a confounding factor in the comparison between MS group 3 and healthy controls, but obviously not in the comparison between MS group 2 and controls. Second, in contrast to the study by Goldman et al. in which the participants had to walk a distance of 175 feet between cones or to the study by Burschka et al. in which the participants had to walk a distance of 20 m between cones, we chose a shorter (25 feet) distance (space issue, possible to do in clinical practice). As a result, participants change their direction and thus, their walking speed more often. However, since we excluded the first and the last 25 feet distance, as well as the first and the last 2.5 m of each 31 feet distance between the cones from the analysis acceleration and deceleration effects can be neglected.

## Conclusions

Wearable inertial sensors are sensitive to differentiate patients in the early stages of MS, in which the EDSS may not provide information about deviations in walking ability. Further, the linear trend measured using inertial sensors could serve as a surrogate parameter of motor fatigue. If it can be shown in future studies that these parameters obtained by means of wearable inertial sensors show sufficient test-retest reliability in MS, we recommend that classic timed walking tests in routine clinical practice should be replaced by readily and automatically applicable gait assessments.

## Data Availability

Data that support the findings of this study are available upon reasonable request from the investigators of the Klinikum Bayreuth GmbH.

## Abbreviations

CIS: Clinically isolated syndrome
EDSS: Expanded Disability Status Scale
MS: Multiple sclerosis
MTC: Minimum toe-to-floor distance
6-min 25-ft walk: 6-minute 25-foot walk
